# Blockchain-based cryptographic framework for secure data transmission in IoT edge environments using ECaps-Net

**DOI:** 10.1038/s41598-025-30906-5

**Published:** 2025-12-05

**Authors:** Islabudeen Mohamed Meerasha, Jafar Ali Ibrahim Syed Masood, Thanapal P, Arumuga Arun R

**Affiliations:** 1 School of Computer Science and Engineering, Vellore Institute of Technology, Vellore, Tamil Nadu India; 2 School of Computer Science Engineering and Information Systems, Vellore Institute of Technology, Vellore, Tamil Nadu India

**Keywords:** Edge computing, Intrusion detection, Internet of things, Secure data transmission, Enhanced capsule network, Squeeze and excitation, Blockchain, Merkle-damgard cryptographic algorithm, Engineering, Mathematics and computing

## Abstract

In the evolving landscape of Internet of Things (IoT), the integration of interconnected devices and cloud computing has revolutionized data collection and processing. However, this connectivity poses numerous security challenges about data privacy, integrity, and security. Traditional cloud-based security approaches inadequate for managing the distributed and dynamic nature of IoT ecosystems. The emergence of the edge computing paradigm allowed for the transfer of data processing and storage closer to local edge devices, but introduces new vulnerabilities at the edges. Thus, an Intrusion Detection System (IDS) is required in this situation. IDS built at the edge can quickly detect and mitigate possible attacks by continually monitoring network traffic, device interactions, and real-time anomalies. Therefore, in this study, we propose an Enhanced Deep Learning (DL)-based IDS integrated with a Blockchain-Based Cryptographic-Algorithm to ensure secure data transmission in an IoT edge computing environment. Initially, the intrusion dataset undergoes preprocessing step to enhance its quality by eliminating unnecessary data and normalizing the dataset. then, the pre-processed data is classified using an Enhanced Capsule Network (ECaps-Net), which incorporates a Squeeze and Excitation (SE) block to highlight important features and surpasses less important ones. After classification, the classified normal data is converted into blocks using Blockchain technology. Every block is hashed using the Merkle-Damgard cryptographic algorithm to ensure data integrity and confidentiality. The proposed framework outperformed existing methods with a maximum accuracy of 98.90% and 98.78% on the KDD Cup-99 and UNSW-NB 15 datasets, respectively. The proposed mechanism protects cloud server and edge devices from malicious access, offering a reliable and efficient solution for secure data transmission in IoT edge environments.

## Introduction

In the modern era, the IoT is defined as a networked system that enables data transmission across multiple interconnected devices via the Internet^1^. The integration of IoT devices and cloud computing has transformed how data is collected, processed, and utilized. However, the rapid proliferation of IoT devices connected to centralized cloud services has raised serious concerns regarding data privacy, integrity, and security. Ensuring that only authorized users can access to trustworthy data has become a critical challenge. The cloud computing paradigm allows users access to robust remote and networked computer resources, including processing and storage capabilities, saving them cost on planning, acquiring, and maintaining these resources^3^. Despite these advantages, the recent broad growth in IoT applications and the exceptionally demanding requirements of contemporary user applications have exposed limitations in the traditional cloud computing model to these new demands^2^. Traditional cloud-based security measures are struggle to manage the dynamic and distributed nature of IoT ecosystems, and they face challenges in processing and securing the vast volumes of data generated by the IoT devices.

To overcome these challenges, a common trend is the adoption of edge computing within IoT frameworks^4^. The edge computing moving processing, management resources, and storage closer to the data source, alleviating latency and bandwidth constraints while enabling real-time processing capabilities. Despite its advantages, edge computing brings forth its own security concerns, especially at the edge where devices are more susceptible to malicious attacks and breaches. Many security and privacy issues previously associated with cloud computing are now shifting to the edge level^5,6^. As a result, IDS have become essential for securing edge environments. Developing IDS at the edge enables real-time monitoring of device behavior and system traffic to detect anomalies or malicious activity^7,8^. However, Conventional IDS solutions often struggle to meet these demands due to limitations in handling the scale and heterogeneity of IoT systems. Conversely, IDS employ an anomaly-based strategy built on learning techniques. Numerous Machine Learning (ML)-based methods, such as support vector machines (SVMs), decision trees (DT), association rule mining, and clustering approaches have been developed for IDS are employed^9^.

Because of their shallow frameworks, these ML approaches are not appropriate for identifying malicious activities. In edge computing environments, the challenge becomes more complex. Because edge computing is distributed lead to the generation of significant amount of noisy data, making it difficult for conventional ML techniques to identify attacks from this data^10–12^. To increase performance, DL algorithms have recently been used to a variety of IDS^13–15^. However, DL-based methods necessitate a significant amount of storage and processing power for data gathering. Furthermore, the focus of many existing models remains only on intrusion detection, often overlooking the critical aspect of secure data transmission. Although, recent studies have explored secure data transfer mechanism. However, it did not resolve a security issue and produce better results in terms of processing speed, data integrity, or confidentiality rate. Thus, secure data transmission remains a pressing concern. Our study tackles this challenge by proposing a novel improved DL-based IDS integrated with a cryptographic scheme to ensure safe data transfer in IoT edge computing environments.

### Motivation

The rapid growth of IoT and the adoption of edge computing have enabled efficient data processing, storage, and management closer to data sources. However, this architectural shift also introduces new privacy and security concerns at the edge architecture, where devices are more susceptible to malicious attacks. Existing security models struggle to adapt to the decentralized and dynamic nature of these systems. Many IDS either lack the accuracy to detect complex attacks or fail to secure data during data transmission. This work is motivated by the urgent need for a security mechanism that not only detects intrusions effectively but also guarantees secure data transfer in IoT edge environment. To achieve this, we propose a solution that combines improved DL-based IDS with a cryptographic scheme to ensure both accurate detection and secure data transmission.

### Contribution of this work

This study presents a securing data transmission framework for IoT edge computing environment by integrating an enhanced DL-based model with a cryptographic scheme. The main contributions of this work are listed below:

- We propose a novel ECaps-Net model integrated with cryptographic scheme to ensure secure data transmission in IoT edge environments.
- The intrusion dataset is collected from publicly available dataset such as, KDD Cup-99 and UNSW-NB 15 dataset.
- Initially, the collected intrusion dataset undergoes preprocessing step to improve its quality by removing irrelevant data and normalizing the dataset.
- The proposed ECaps-Net model is used to classify the incoming data as either normal data or intruded data. ECaps-Net incorporates a SE block into the conventional CapsNet, which helps to highlight significant features while suppressing irrelevant features, thereby improving the accuracy of the classification performance.
- To ensure secure data transmission, the classified normal data is converted into data blocks using blockchain technology.
- Each block is hashed using the one-way compression function based on the Merkle-Damgard cryptographic algorithm to ensure data integrity and prevent tampering.
- The effectiveness of the proposed framework is evaluated based on various metrics such as accuracy, recall, F-score, sensitivity, precision, processing time, confidentiality rate, and data integrity rate.

### Organization of the paper

The structure of the remaining sections is as follows: The relevant research on safe data transfer in IoT edge computing is reviewed in Section 2. The suggested method for safe data transfer in an edge computing setting is presented in Section 3. Section 4 provides the experimental results and discussion. Final conclusion is described in Section 5.

## Related works

Many researchers developed IDS for edge computing environments. This section examines a select sample of these works. For edge computing (EC) and fog computing (FC) environments, an effective seeker optimization technique combined with ML-enabled IDS (ESOML-IDS) was presented by Alzubi et al.^16^. To find the best feature set for intrusion detection in EC and FC scenarios, the ESOML-IDS framework specifically uses a novel ESO-based feature selection technique. To improve intrusion detection capabilities, the authors also used a Denoising Autoencoder (DAE) in conjunction with a complete learning particle swarm optimization (CLPSO) approach. According to the evaluation results, the ESOML-IDS model performed better than current approaches in terms of accuracy, precision, F1 score, and recall. Still, the model has trouble accurately identifying intrusions in fog and edge computing contexts.

For intrusion detection in a mobile edge computing context, Jiao et al.^17^ developed a model called XGBoost-TCN, which combines Extreme Gradient Boosting Decision Tree with Temporal Convolutional Network. The approach used the XGBoost algorithm to reduce high-dimensional traffic data to lower dimensions, followed by the application of a TCN model to detect abnormal traffic patterns. The performance and adaptability of the method were evaluated on a public dataset, performance evaluations showed improved detection accuracy. However, the technique did not achieve optimal computational efficiency.

A hierarchical blockchain-based federated learning (FL) architecture was created by Mohanad Sarhan et al.^18^ to facilitate safe and private collaborative IoT intrusion detection. A hierarchical FL design was used by the designed ML-based IDS to protect organizational data and the learning process. The processes and transactions (model modifications) would operate on a safe unchangeable ledger, and the smart contract would confirm that the tasks were completed correctly. The framework was reliable, highly effective, and robust in maintaining the integrity of IoT networks. However, they did not incorporate k-means clustering in combiners to improve adversary detection.

Sun H et al.^19^ introduced a combined framework that integrated transformer and neural network models to address data imbalance issues in network traffic, which impacted network intrusion detection performance. First, Tomek Links, SMOTE, and WGAN were used to preprocess the data to solve the class-imbalance problem. Second, the transformer was used to encode traffic data to extract the correlation between network traffic. Finally, a hybrid DL network model combining a bidirectional Gated Recurrent Unit (GRU) and deep neural network (DNN) was created. A DNN was used to extract deep level features, and softmax is used to complete classification. Experiments were conducted on the NSLKDD, UNSWNB-15, and CICIDS2017 datasets, demonstrating improved detection accuracy. The experimental results highlighted enhanced communication security for network data. However, the framework did not manage to achieve optimal computational efficiency.

In order to tackle security issues, Fenanir, S., Semchedine, F. et al.^20^ presented various kinds of smart intrusion detection (SID) methodologies, mostly based on ML and DL approaches. The study used FL to solve privacy and data security issues. Three recognized IoT datasets and three well-known DL models were used to assess the efficacy of this strategy. The results demonstrated robust accuracy in detecting intrusions within IoT environments. However, the approach did not succeed in achieving high processing power.

In order to detect intrusive traffic in the MEC environment, Singh et al.^21^ developed an edge-based hybrid IDF (EHIDF) architecture utilizing a ML technique. This framework was made up of many classifiers and detecting modules. The Meta-AdaboostM1 algorithm was used by the Hybrid Detection Module (HDM). To examine the edge-based IDF’s security strength, a game theoretical method was used. This architecture achieved high accuracy and the capacity to identify unknown or novel assaults. The EHIDF successfully resolved current detection problems by identifying new, unidentified assaults with a low false alarm rate (FAR). While the findings showed improved performance, the framework was unable to achieve efficiency and scalability.

CNN-based IDS were developed by Haq et al.^22^ for the improved data rates for the GSM Evolution (EDGE) computing environment. Events were divided into two categories by the system: attack and non-attack. The study’s findings demonstrated how effective this tactic was. Binary and multiclass classification efforts were undertaken, and the feature vector size was minimized using Principal Component Analysis (PCA) based on feature engineering and extraction. According to the experimental results, DL enabled the method to obtain greater precision. However, it did not leverage other available datasets.

The BFLIDS, Blockchain-enabled FL-based IDS was presented by Hee-Cheol Kim et al.^23^ to improve security in IoMT networks. This method used FL to protect data privacy and blockchain to secure transaction records. To improve model accuracy, they added Kullback-Leibler divergence estimate and adaptive weight computation to the FedAvg algorithm. For classification, Adaptive Max Pooling-based convolutional neural network (CNN) and a modified Bidirectional Long Short-Term Memory (BiLSTM) with attention and residual connections were utilized on Edge-IIoTSet and TON-IoT datasets. The BFLIDS improved the security and privacy of IoMT networks by successfully detecting intrusions. However, the framework lacked the incorporation of advanced DL techniques within the FL paradigm, which limited its ability to model complex inter-device relationships and detect sophisticated, coordinated intrusion patterns.

Meanwhile, the above methods mainly identify intrusions in incoming data; do not address the safety of data transmission in IoT edge computing environments. Therefore, the proposed work overcomes the above problems by introducing an Enhanced DL-based model integrated with Blockchain-based Merkle-Damgard Cryptographic algorithm for secure data transmission in IoT edge environments.

## Proposed methodology

In the evolving landscape of the IoT, the integration of cloud computing and networked devices has transformed data collection, analysis, and utilization. With the quick advancement of sensor devices and their incorporation into the IoT, security becomes crucial to granting authorized users access to trustworthy data. The majority of enterprise security solutions in use today are cloud-based, with service providers handling all security needs. Traditional cloud-based security approaches struggle to manage the dynamic and distributed nature of IoT ecosystems. Edge computing has emerged as an innovative paradigm for addressing these challenges, enabling data processing closer to the data source. The primary goal of edge-based security solutions is to manage security needs directly at the network’s edge, reducing latency and enhancing responsiveness. Nevertheless, this change in architecture places the privacy and security issues on the edge architecture. As a result, implementing effective intrusion detection in such decentralized environments becomes increasingly complex. IDS are essential for improving the security posture of IoT edge computing infrastructures by continuously monitoring network behavior and identifying potential threats.

**Fig. 1:**
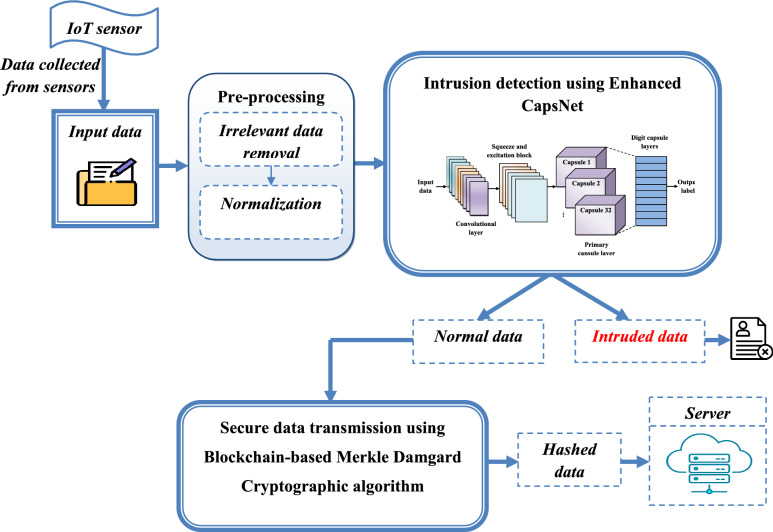
Overall architecture of the proposed secure data transmission framework in IoT edge computing.

Therefore, we propose a novel framework for secure data transmission in IoT edge computing environments by combining ECapsNet-based IDS with blockchain technology. Initially, the intrusion dataset undergoes preprocessing step to improve its quality by removing irrelevant data and normalizing the dataset. Next, the preprocessed input data is classified using ECapsNet. ECapsNet is a variation of the traditional CapsNet that incorporates a SE block. This enhancement enhances the accuracy of the classification task by highlighting important features and suppressing irrelevant features. Once the data is classified, additional security measures are applied to the normal data to ensure confidentiality and integrity during transmission and storage. We employ a blockchain based on the merkle-damgard cryptography algorithm for safe data transfer. The classified normal data is converted into data blocks using blockchain technology. Utilizing the merkle-damgard cryptographic algorithm, the one-way compression function generates a hash for every block, guaranteeing data security and integrity. The hashed data are then securely transmitted and kept on the server. The fundamental structure of the suggested technique for secure data transmission in IoT edge computing is shown in Figure 1. The subsequent sections provide a detailed explanation of each step in the proposed methodology.

### Pre-processing

Initially, the intrusion dataset is collected and preprocessed step is performed to improve its quality by removing irrelevant data and normalizing the dataset.

#### Remove irrelevant data

The values of invalid or non-informative attributes, including infinity and NaN are eliminated. This step ensures the efficient execution of the model by eliminating redundant data.

#### Normalization

The dataset contains features with varying maximum and minimum values, which can affect model performance. Normalizing all values in the range [0, 1] improves the classifier’s efficiency. Min-max normalization is utilized to transform attribute ranges to be within the range [0.1]. Equation (1) provides a formula for min-max scaling:

1 $$Y_{norm} = \frac{{Y - Y_{\min } }}{{Y_{\max } - Y_{\min } }}$$

Where, $Y_{norm}$ denotes the normalized attribute value, $Y$ denotes the original attribute value, $Y_{\max }$ and $Y_{\min }$ denotes a maximum and minimum values of the attribute. By applying these preprocessing steps, the dataset is cleaned and standardized for improved model performance in further classification process.

### Intrusion detection using enhanced CapsNet

ECaps-Net is proposed to classify if the input preprocessed data is malicious or normal. To improve the classification performance, a SE block is added to the traditional CapsNet is termed as ECapsNet. This aims to highlight significant features and minimize the effect of less important ones. CapsNet is the most recent advancement in DL networks, designed to address drawbacks of the conventional CNN methodology^24^. A capsule is a made up of several organized neurons, each of which represents a different attribute of a certain item. A capsule’s instantiation parameter is represented by each neuron. The capsule’s dimension is equal to the number of neurons. The possibility that a certain object exists is represented by the length of the capsule.

**Fig. 2:**
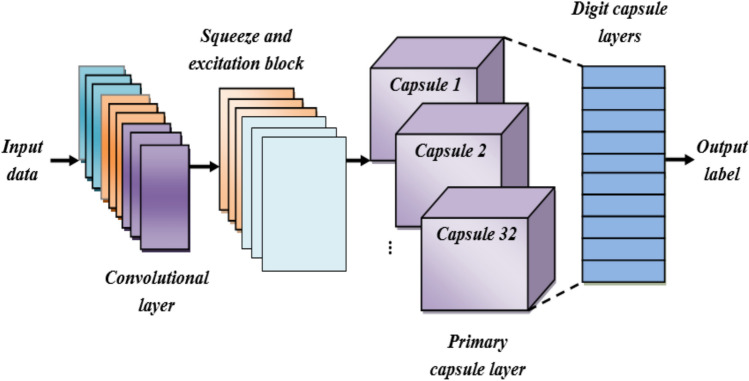
The structure of the proposed enhanced CapsNet.

Convolutional layer (CL), SE block, primary capsule layer (PC), and digit capsule layer (DC) are the four distinct layer types that make up the ECaps-Net model. The convolution layer produces a local feature map by capturing features from the input data using a convolutional filter. The SE block uses squeeze and excitation operations to carry out feature recalibration process. The spatial correlations between the features are captured by the primary capsule layer. The feature map is transformed from scalars into vectors by the primary capsules. Eight-dimensional, 32 different 6 × 6 capsules convert the scalar data into vectors with direction information. The DC layer consists of ten fully connected (FC) capsules, each of which can be expressed by a 16-dimensional vector. Using a dynamic routing (DR) algorithm, the DC layer anticipates low-level features that are encoded by the PC layer. The coupling coefficient value of low layer capsule and the matching high layer capsule are adjusted by the dynamic routing algorithm based on their resemblance, the greater the similarity, the larger the coupling coefficient among them. In the last layer, the length of every capsule is calculated to determine the likelihood that the entity exists, which is effectively the likelihood that the labelling result is valid. The general architecture of the suggested ECaps-Net IDS is shown in Figure 2. The suggested ECaps-Net model is explained mathematically in the following.

#### Convolutional layer

The convolutional process extracts low-level characteristics from the input data, which uses various filters in the CL. Assume that $X_{i} \in R$ is the input data. $X^{j} = [x_{1}^{j} ,x_{2}^{j} ,....,x_{n}^{j} ]$_,_$j = 1,2,...,M$ is the representation of the input data vector $X^{j}$. A filter $W^{j} \in R^{n}$ is used in a convolution operation, where it is utilized to a vector $X^{j}$ to create a new feature. As an example, the vector $X^{j}$ can be used to generate the feature $Y_{i}^{j}$ by:

2 $$Y_{i}^{j} = f(W_{{}}^{j} .X^{j} + B^{j} )$$

In this case, $f$ is a non-linear activation function and $B^{j}$ represents a bias factor. To create a feature map, the filter $W^{j}$ is used to each vector $X^{j}$,here $j$ denotes number of vectors.

3 $$Y^{j} = [y_{1}^{j} ,y_{2}^{j} ,y_{3}^{j} ,...,y_{n}^{j} ]$$

#### SE block

**Fig. 3:**
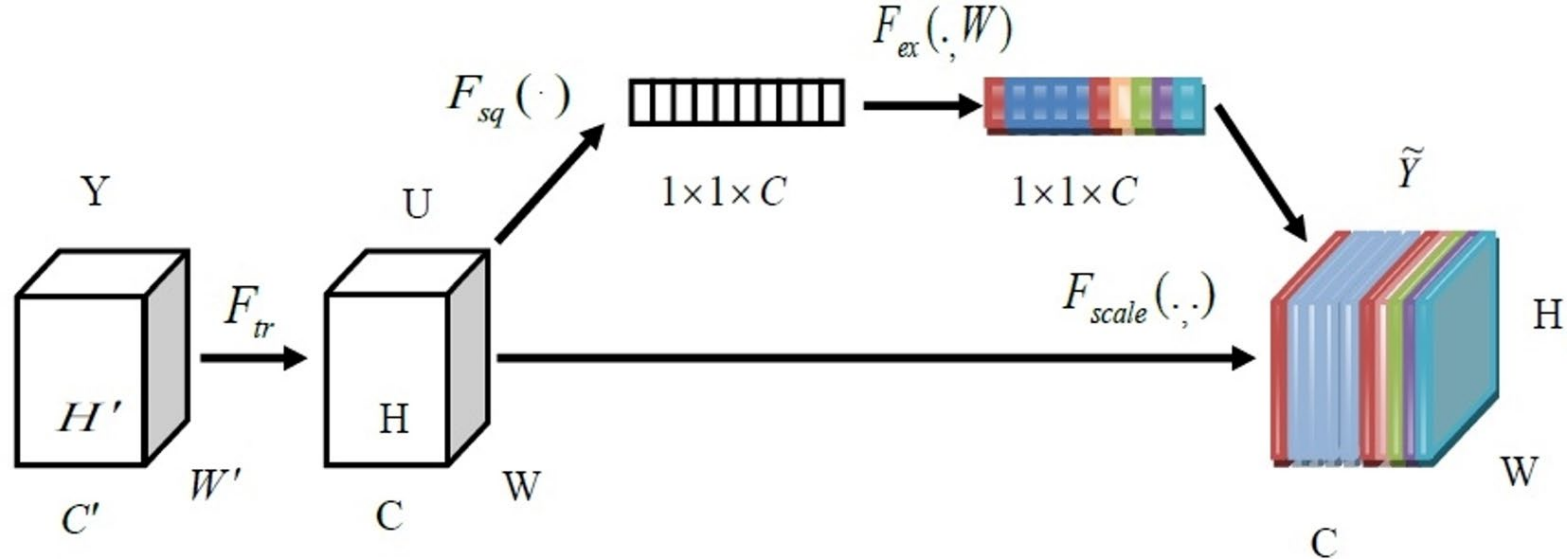
The structure of the SE block.

We include SE block in ECaps-Net, which recalibrates deep feature maps generated by convolutional layer, to improve the effectiveness of classification method. SE processes allow SE blocks to automatically learn global data, eliminate redundant data, and pick targets based on important attributes by applying various weight ratios to filtering channels. Figure 3 displays a structure that depicts the composition of a SE block.

With a dimension of (W′, H′, and C′) as input, the supplied data $Y$ can be converted through a sequence of convolutional transformations $F{}_{tr}$ and mapped to the feature map U as $U \in R^{H \times W \times C}$. The result $U = [u_{1} ,u_{2} ,....,u_{C} ]$ could be expressed below:

4 $$u_{c} = v_{c} *y = \sum\limits_{s = 1}^{{C^{\prime}}} {v_{C}^{s} *y^{s} }$$

Where $V = [v_{1} ,v_{2} ,...v_{C} ]$ indicates a learnt convolution kernels; and $v_{c} = [{v}_c^1, {v}_c^2,...,{v}_c^c ]$ indicates a parameters of the ${C}^{th}$ filter; * denotes a convolution operation; $y = \left[ {y^{1} ,y^{2} ,...,y^{{c^{\prime}}} } \right]$ and $y^{s}$ represents s^th^ input, $u_{c} \in R^{H \times W}$.

#### Squeeze operation

Squeeze operations are carried out using global average pooling (GAP), which captures dependencies between channels. The squeeze transformation $F_{sq}$ then converts feature mappings $U$ into single-dimensional, global spatial feature vectors, using a statistic $z \in R^{C}$ generated by compressing $U$ along the spatial dimension $H \times W$_described below_:

5 $$z_{c} = F_{sq} (u_{c} ) = \frac{1}{H \times W}\sum\limits_{p = 1}^{H} {\sum\limits_{q = 1}^{W} {u_{c} (p,q)} }$$

#### Excitation operation

To utilize the information gathered during the squeeze operation, a subsequent operation is performed to effectively record channel-wise dependencies. Based on learning parameters that explicitly characterize the correlation between feature channels, the excitation operation creates weights for every feature channel. Therefore, use double completely connected layers and the self-gating technique to adaptively recalibrate feature maps. It might be expressed like this:

6 $$s = F_{ex} (z,W) = \sigma (g(z,W)) = \sigma (W_{2} \delta (W_{1} ,z))$$

Where $\delta$ denotes a ReLU activation function, $\sigma$ indicates a sigmoid function,$W_{1} \in R^{{{C \mathord{\left/ {\vphantom {C r}} \right. \kern-0pt} r} \times C}}$ and $W_{2} \in R^{{{C \mathord{\left/ {\vphantom {C r}} \right. \kern-0pt} r} \times C}}$, and r represents a dimensionality reduction ratio. Using the activations $s$, U is finally rescaled to yield the results of the SE block.

7 $$\tilde{y}_{C} = F_{scale} (u_{c} ,s_{c} ) = s_{c} .u_{c}$$

Here,$\tilde{y} = [\tilde{y}_{1} ,\tilde{y}_{2} ,....,\tilde{y}_{c} ]$ and $F_{scale} (u_{c} ,s_{c} )$ denotes channel-wise scalar multiplication $s_{c}$ and feature map $u_{c} \in R^{H \times W}$.

#### Primary capsule layer

The recalibrated feature maps from the SE block are sent into a primary capsule layer. The spatial correlations between the features are captured by this PC layer. Each of the 32 capsules $i$ in the PC layer contains an activity vector $y_{i}$ that encodes the spatial information as instantiation parameters. Each capsule’s distinct concepts are identified using the trainable weight ($W$) of DR. In this case, $j \in [1,\,N_{class} ]$ denotes an16-dimensional output capsule index,$i \in [1,\,N_{PC} ]$ denotes an index of the initial 8-dimensional capsule of dimensions, and the dimension of $w_{ij}$ assumed to be $8 \times 16$. Taking $y_{i}$ as the output of capsule $i$, the following calculation is made to predict it for the primary capsule $j$:

8 $$\hat{y}_{j|i} = w_{ij} y_{i}$$

Here, $w_{ij}$ is the weighting matrix and $\hat{y}_{j|i}$ represents a predicting vector of the higher-level output of the $j$ ^th^ capsule, which is determined by *i* the primary capsule layer’s capsule.

#### Dynamic routing

The output capsules are extracted from the original capsules via dynamic routing. The DR algorithm modifies coupling coefficient values between the low-layer capsule and the corresponding high-layer capsule according to their degree of similarity; the higher the coupling coefficient between them. $y_{i}$ represents the $i$ ^th^ initial capsule. For every main capsule $i$, a yield block of structure $N_{class} \times 16$ is supplied. Routing weights $b$, other kind of weight of dimension $N_{PC} \times N_{class}$, are taken into account for the operation of DR. The outcome capsules are constructed by combining individual concepts with them. Unlike $W$, these weights learn through further iterations of dynamic routing that are depend on the connection between principles and overall outcomes. These weights are set to 0 at the beginning of every forward pass. The following softmax function, provided by equation (9), can be used to compute the coupling coefficients $ce_{ij}$.

9 $$ce_{ij} = \frac{{\exp onent(b_{ij} )}}{{\sum\nolimits_{k} {} \exp onent(b_{ik} )}}$$

In this case, if capsules $i$ and $j$ should be connected, the log likelihood of this happening is represented by $b_{ij}$.

In order to create combined output capsules, the individual concepts $\hat{y}_{j|i}$ will be joined using this coupling coefficient. Squashing $sq{}_{j}$ will yield the $j$ ^th^ coupled outcome capsules, as shown in equation (10).

10 $$sq_{j} = \sum\limits_{i} {ce_{ij} } ,\hat{y}_{j|i} = w_{ij} y{}_{i}$$

#### Output capsule layer (Digit capsule layer)

A non-linear squashing function will be utilized to guarantee that the smaller vectors are compressed to nearly zero length and the larger vectors are compressed to a length less than one. Then outcome capsule $o_{j}$ is shown in equation (11).

11 $$o_{j} = \frac{{||sq_{j} ||^{2} }}{{1 + ||sq_{j} ||^{2} }}.\frac{{sq_{j} }}{{||sq_{j} ||}}$$

Where $sq_{j}$ represents capsule $j$‘s input vector.

The agreement among the separate output capsules ($\hat{y}_{j|i}$) and the squashed aggregate result capsules ($o_{j}$) is computed utilizing a simple dot product. In this case, singular capsules that concur with the overall results will be prioritized. To do this, modify the $b_{ij}$ as per equation (12).

12 $$b_{ij} = b_{ij} + \hat{y}_{j|i} .o_{j}$$

#### Loss function

This will separate into two main categories: the object existence margin losses for a data created from mean square losses and the output capsule. The formula in equation (13) will be used to compute the object’s marginal losses.

13 $$l_{k} = T_{k} \max (0,m^{ + } - ||o_{k} ||)^{2} + \lambda (1 - T_{k} )\max (0,||o_{k} || - m^{\_} )^{2}$$

In this case, when class $k$ is denote $T_{k}$ is 1 and 0 otherwise. The hyperparameters that must be learned throughout the training procedure are terms, $m^{ + }$, $m^{\_}$, and $\lambda$.

#### Decoder network for regularization

To encourage the digital capsule to encode the input number’s instantiation parameters, reconstruction loss is applied at the network end. Only the digital capsules that generate the proper forecast are used to recreate the input data during training; any vectors that do not yield the correct prediction are set to zero. To reduce the total squared differences between the pertinent pixels of the input data and the reconstructed data, the digital capsule’s output is routed to a decoder made up of three FC layers. Equation (14) provides the reconstruction loss.

14 $$R = MSEloss(I,I^{\prime})$$

Here,$I^{\prime}$ denotes an input data,$I$ stands for the reconstructed data. Equation (15) provides an approximation of the net losses.

15 $$L_{k} = m_{k} + \alpha R$$

In this instance,$\alpha$ indicates the downward scaling parameter. It prevents the losses from reform from surpassing the losses from the border. Algorithm 1 shows the pseudo-code of the proposed ECapsNet model.

**Algorithm 1:**
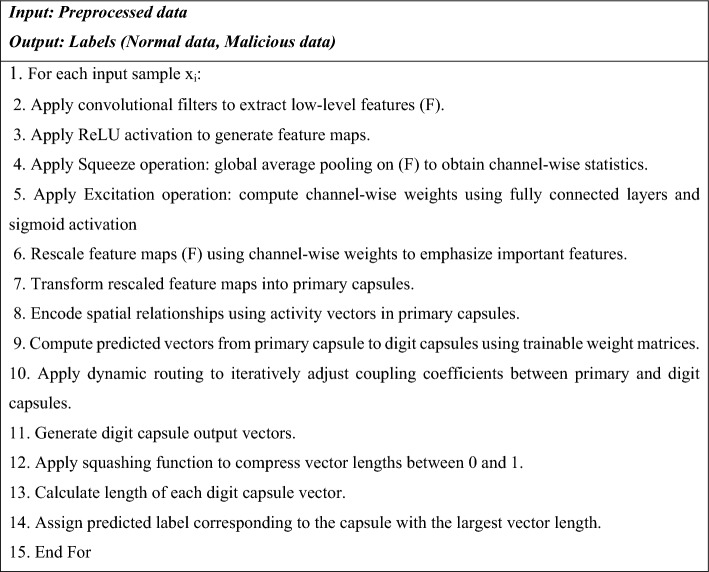
Pseudo-code of the proposed ECaps-net model

By using capsule network and SE block, the proposed ECapsNet effectively classifies the incoming data normal or malicious. By its extensive architecture and integrating a Squeeze and Excitation (SE) block into the traditional CapsNet, it emphasizing important features while decreasing the impact of less important ones, significantly improving intrusion detection classification performance. The dynamic routing algorithm within the E-capsule network ensures that spatial relationships and hierarchical structure are preserved, which is crucial for accurate classification of intrusion detection system. By using dynamic routing algorithm and SE block, effectively enhances the detection and classification of data as normal or intruded with improved accuracy. Once classified, the normal data proceed to the next step for secure data storage.

### Secure data transmission using merkle-damgard cryptographic algorithm

Following the classification of input data into malicious and normal, additional security measures are implemented in place to guarantee the confidentiality and integrity of normal data during transmission and storage. The merkle-damgard cryptographic hash-based blockchain technology is used to ensure secure data transmission. For secure transmission of data, the classified normal data is divided into blocks via block chain technology. To ensure secure data transmission, the merkle-damgard cryptographic algorithm is used to create a hash for every data block, and added to the blockchain, making tampering infeasible. To protect data transmission from unauthorized parties, blockchain technology is used.

A blockchain, which is based on the Bitcoin protocol, is a distributed transaction database made up of every node^30^. Blockchain is a distributed, decentralized network that provides immutability, security, privacy, and transparency, which are crucial in preventing unauthorized data modifications and ensuring that transmitted data remains untampered^28,29^. All transactions on the Blockchain are thought to be completely safe and verifiable, even though there is no central authority to approve and verify them. Only the consensus mechanism, a crucial part of all blockchain networks, makes this possible. A consensus algorithm is a process that allows all of the Blockchain network’s peers to agree on the distributed ledger’s current state. Consensus techniques ensure reliability in the Blockchain network and foster trust amongst unknown peers in a distributed computing setting. The consensus process basically verifies that every new block that is added to the Blockchain represents a single version of the truth that all of the nodes in the Blockchain agree upon. A mining algorithm, which is the set of guidelines or instructions a system adheres to in order to produce a legitimate block, is also a crucial part of blockchain technology. Blockchain technology, which underpins cryptocurrencies such as Bitcoin, Tether, Ethereum, Dogecoin, etc., enables secure and verifiable data transactions through the use of a decentralized control system and cryptographic mechanisms^27^. These foundational principles are now being applied in secure data transmission systems like IoT edge environments.

In this work, we adopted Hyperledger Fabric-based private blockchain technology. Hyperledger Fabric offers a lightweight design with support for customizable consensus protocols, low-latency communication, and fine-grained access control-all of which are essential in resource-constrained IoT settings. Its permissioned nature ensures that only authenticated devices participate in the network, aligning with the proposed framework’s objective of secure data transmission. The Hyperledger Fabric architecture uses a Practical Byzantine Fault Tolerance (PBFT) consensus algorithm to ensure consistency and reliability among participating IoT-edge nodes. PBFT has been selected due to its efficiency and applicability to permissioned environments, guaranteeing all authenticated nodes agree on a single valid version of the distributed ledger even when faulty or compromised devices are involved. The consensus process employs a sequence of pre-prepare, prepare, and commit steps to facilitate fast block validation without employing computationally expensive mining utilized in public blockchains like Bitcoin. Through the reduction of redundant communication overhead as well as deterministic finality, PBFT facilitates low-latency confirmation of blocks and high throughput, which are essential in real-time IoT-edge systems. Hyperledger Fabric inherently supports smart contracts (referred to as chain-code), which define the business logic and validation rules for data access and storage.

In the proposed framework, smart contracts enforce access permissions and specify transaction endorsement policies, ensuring that only authorized IoT devices can execute write operations or retrieve hashed data. Endorsement policies define which nodes must validate a transaction before it is committed, providing additional layers of authentication and trust.

This mechanism guarantees secure data access, integrity verification, and transparency throughout the blockchain network. In addition, the integration of PBFT and Merkle–Damgård hash structure improves data integrity and tamper resistance, keeping block propagation secure and synchronized within the network. This design achieves fault tolerance, scalability, and energy efficiency while keeping the blockchain trustworthy under multi-device, resource-limited environments.

**Fig. 4:**
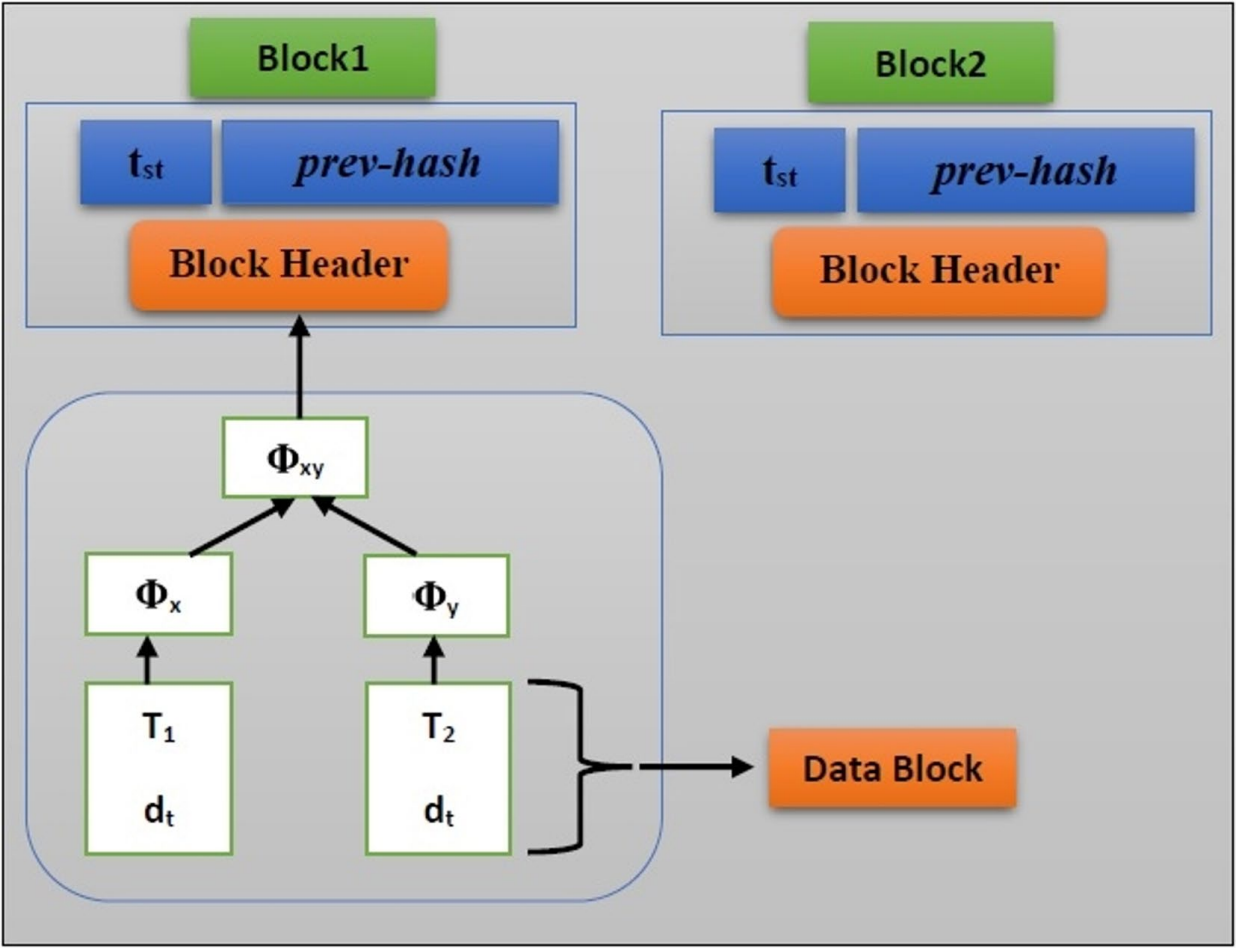
The structure of the blockchain.

Figure 4 shows the blockchain’s structure, which consists of separate blocks connected to create a chain. Every block in the chain consists of data $d_{i}$, a time steps $(t_{st} )$, and cryptographic hash of prior block $(prev - hash)$. Every user on the blockchain has a unique transaction history. The sensitive data gathered by sensor nodes is included in every transaction. Unauthorized parties cannot access the data while it is being sent from the source to the server. The data is protected by the Merkle-Damgard hash cryptography algorithm. Every transaction in a block chain is built using the merkle-damgard hash cryptographic algorithm. The data is separated into message blocks. The hash value is created for every message block. The server receives sensitive data with its final hash value. As a result, data security is enhanced. For block confirmation, is utilized the hash of the prior block $(prev - hash)$. The block’s creation time is indicated by time steps $(t_{st} )$.

**Fig. 5:**
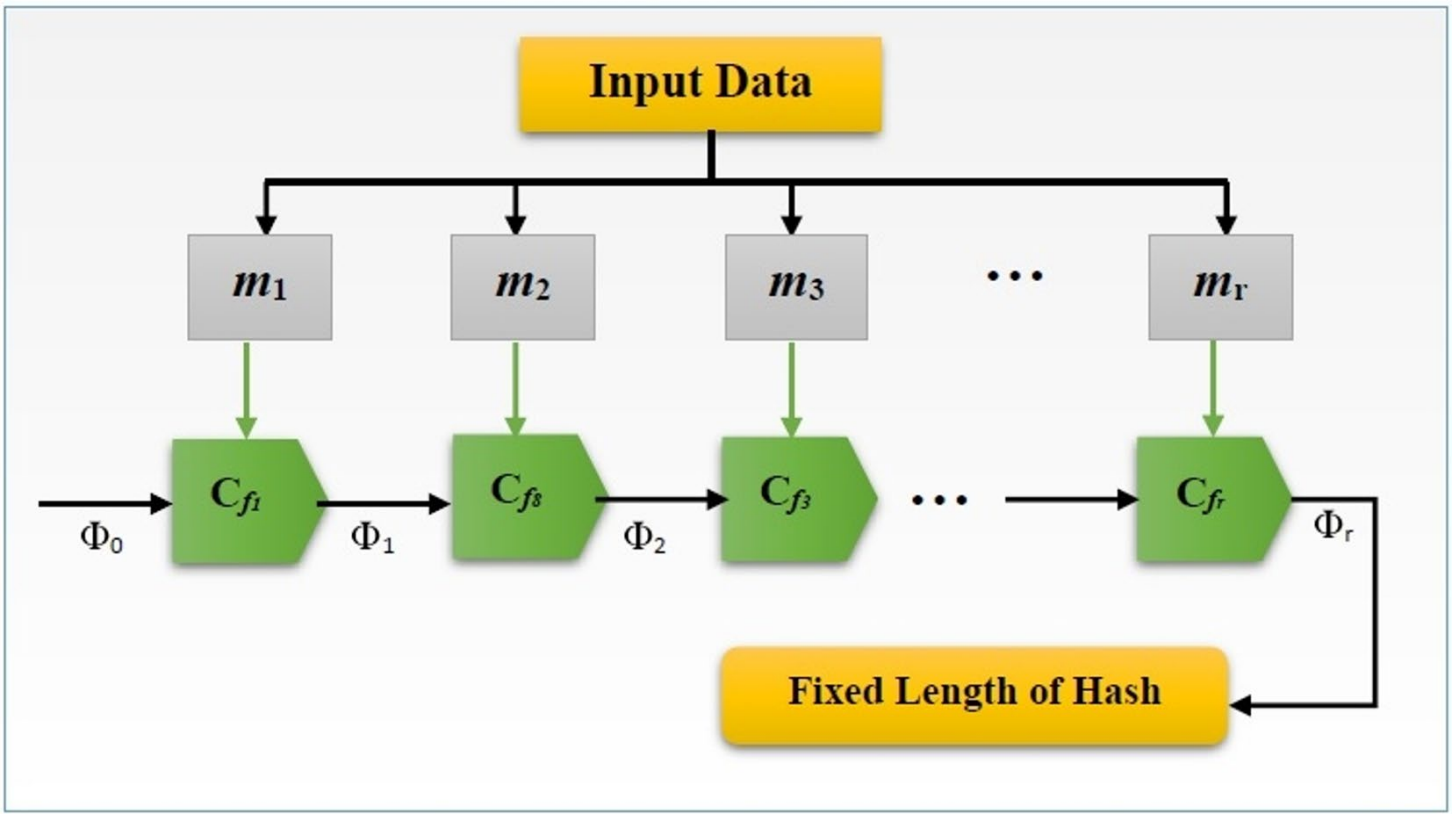
Block diagram of the merkle-damgård constructions.

Merkle-damgard cryptography transforms a message’s length into a fixed-length hash value via a one-way compression mechanism. The hash value, sometimes referred to as the message’s fingerprint, is produced from input data. The hash value can be significantly impacted by even minor changes to the input data. In order to guarantee data integrity for safe data transfer, the Merkle-Damgard hash cryptographic technique is utilized. Figure 5 illustrates how the compression function operates. The merkle-damgard structures’ block diagram in figure 5 showed how a fixed hash is produced for every input piece of data via one-way compression.

Before starting the hash-generating process, the merkle-damgard constructs first split the sensitive data’s input size into several message blocks of a predetermined size.

16 $$d_{sz} \to m_{1} ,m_{2} ,m_{3} ,...,m_{r} {}^{{}}$$

Where $m_{1} ,m_{2} ,m_{3} ,...,m_{r} {}^{{}}$ specifies a block of messages having a set size, and $d_{sz} {}^{{}}$ indicates the input size of the sensitive data. Following data division, the message block is passed to the compression function ($c_{fr}$), which accepts an $r$-bit message block $(m_{r} )$ and an $r$ bit chaining value ($\Phi_{x}$), or final hash.

The produced hash is a $\Phi_{x} \in \{ 0,1\}$ constructed by iterating the compression function $c_{f1} ,c_{f2} ,..,c_{fr}$ to process a message of fixed length using compressive Merkle-Damgård construction.

**Fig. 6:**
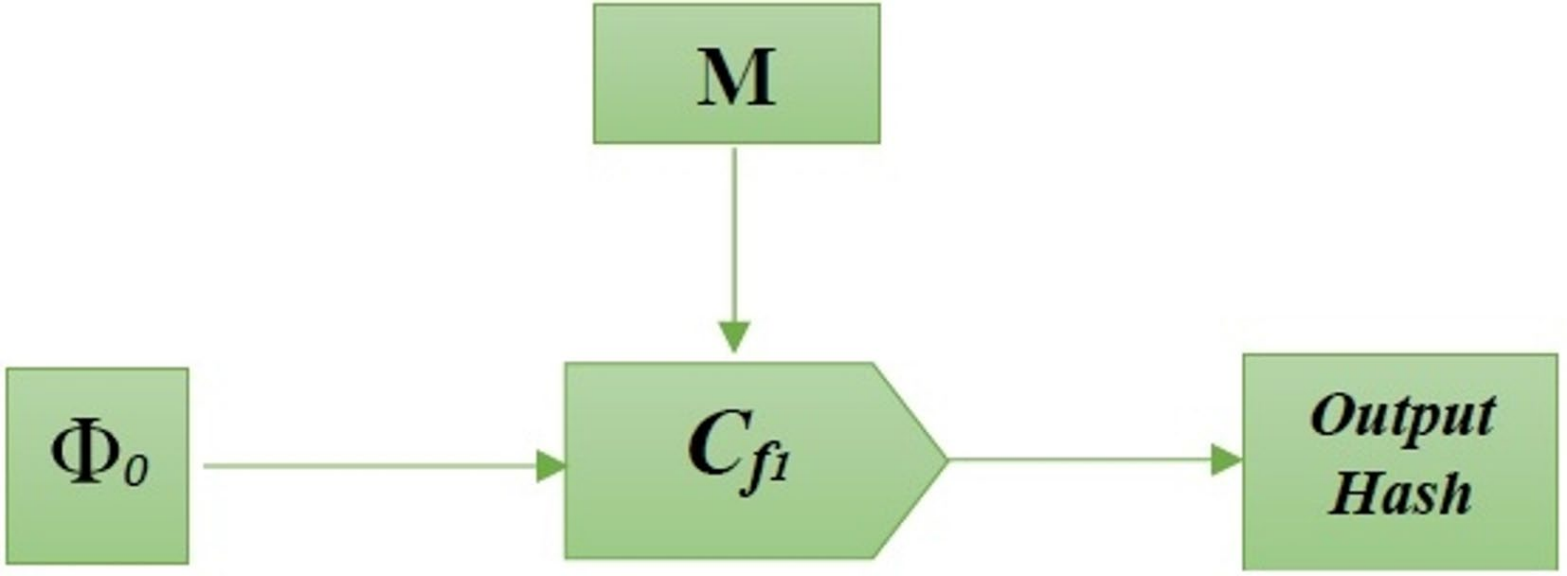
The structure of the compression function.

A simple block cipher technique serves as the compression function. It produces a single output (the hash), which has the same size as the input hash and returns two fixed-size inputs (the message block and the preceding hash). Figure 6 illustrates the compression function’s architecture. $\Phi_{x}$ denotes an initial hash, as seen in figure6.

17 $$\Phi_{x} = c_{f} [\Phi_{i - 1} ,m_{i} ],i = 1,2,3,..,r$$

Where, $\Phi_{x}$ a stands the data’s final hash value, $c{}_{f}$ for the compression function, $\Phi_{i - 1}$ for the previous block’s hash, and $m_{i}$ for the message block. Because of this, only authorized users are able to access data, increasing data confidentiality and integrity. Algorithm 2 shows the pseudocode of Merkle-Damgard Cryptographic algorithm.

**Algorithm 2:**
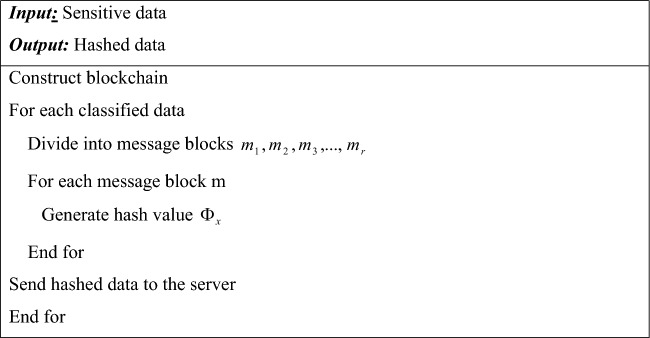
Merkle-damgard cryptographic hash blockchain.

An effective cryptographic mechanism for secure transmission of data is offered by the proposed Merkle-Damgard algorithm. Finally, the combination of the proposed ECapsNet with Merkel-Damgard Cryptographic based blockchain algorithmensures the secure data transmissionin IoT edge computing environments.

## Results and discussion

This part examines the execution of the developed model. Python is used for implementing the suggested method. A Windows-based Intel Core i5 CPU operating at 1.6GHz and 4GB of RAM are necessary for the deployment of the system. We’ve examined two different scenarios: one involves analyzing intrusion detection techniques and the other involves evaluating secure data sharing method. For this investigation, we used the KDD CUP 99 and UNSW-NB 15 dataset. We combine our experimental investigation with state-of-the-art methods using several metrics, including accuracy, precision, recall, sensitivity, f-score, confidentially rate, data integrity rate, processing time, latency, throughput, energy usage, latency, and hash computation time. Table 1 shows the parameter settings of the proposed method.

**Table 1:** parameter settings.

Parameters	Values
Optimizer	Adam
Loss	Mean-squared-error
Batch-size	256
Epochs	10
Activation	ReLU
Learning rate	0.001
Kernel-size	3*3
Dropout rate	0.4
No. of channel in primary capsule layer	32
Dimension of capsule in primary capsule layer	8
Dimension of capsule in digit capsule layer	16
No. of routing iterations	2

### Description of the dataset

The KDD-Cup 99 and the UNSW-NB 15 dataset are the used in this study’s experimental analysis.

#### KDD Cup 99 dataset

For intrusion detection, among the most frequently utilized datasets is the KDD Cup 99^25^.

This collection consists of a large number of network connection records, each classified as normal or as one of numerous forms of attacks. Each record has 41 useful features that describe the attribute of the network connection. Although the data collection does not contain IP addresses, it does offer high-level data, such as the number of unsuccessful login attempts and basic TCP connection data. There are 24 distinct attack types included. Four categories-DoS, Probe, R2L, and U2R-are frequently used to describe these infiltration attempts. Each record in these databases is classified as an attack or a normal.

#### UNSW-NB 15 dataset

The Australian Centre for Cyber Security (ACCS) Cyber Range Lab’s IXIA PerfectStorm application was utilized to create the UNSW-NB 15 data collection^26^. The collection contains nine groups of contemporary assaults. These risks include nonexclusive, misuses, worms, shellcode, DoS, inspection, fuzzers, and secondary transit. There were 49 features in the dataset, which were divided into five categories: basic, content, flow, additional generated and time features. This collection has 2,360,854 records in total. They are divided into 1,914,519 for training and 446335 for testing.

### Ablation study

To evaluate the contribution of the SE block in the proposed ECaps-Net, we conducted an ablation study comparing the model with and without the SE block in terms of accuracy, precision, and recall on the KDD-Cup 99 and UNSW-NB 15 datasets, as shown in table 2. The results indicate that incorporating the SE block into the conventional CapsNet significantly enhances the performance of ECaps-Net across all evaluation metrics. The SE block dynamically recalibrates feature maps by assigning higher weights to important features and suppressing less relevant ones.

**Table 2:** Ablation study results for se block contribution.

Model	Dataset	Accuracy (%)	Precision (%)	Recall (%)
ECaps-Net without SE	KDD-Cup 99	96.54	96.51	96.48
ECaps-Net with SE	KDD-Cup 99	98.90	98.78	98.65
ECaps-Net without SE	UNSW-NB 15	96.32	96.45	96.33
ECaps-Net with SE	UNSW-NB 15	98.78	98.74	98.54

This demonstrates that the SE block is a crucial component of ECaps-Net, contributing substantially to improved feature representation and overall intrusion detection performance in IoT edge computing environments.

### Experimental results

This part examines the outcomes of the proposed method using the UNSW-NB 15 and KDD-Cup 99 datasets. This section examines the training and validation epoch-accuracy and epoch-loss graphs as well as the confusion matrix of suggested technique utilizing the KDD-Cup 99 and UNSW-NB 15 datasets.

**Fig. 7:**
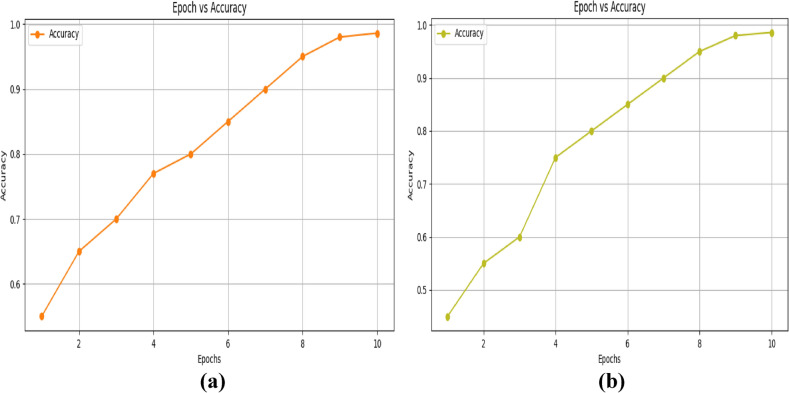
Epoch- accuracy graph for the KDD-Cup 99 dataset (**a**) training and (**b**) validation.

**Fig. 8:**
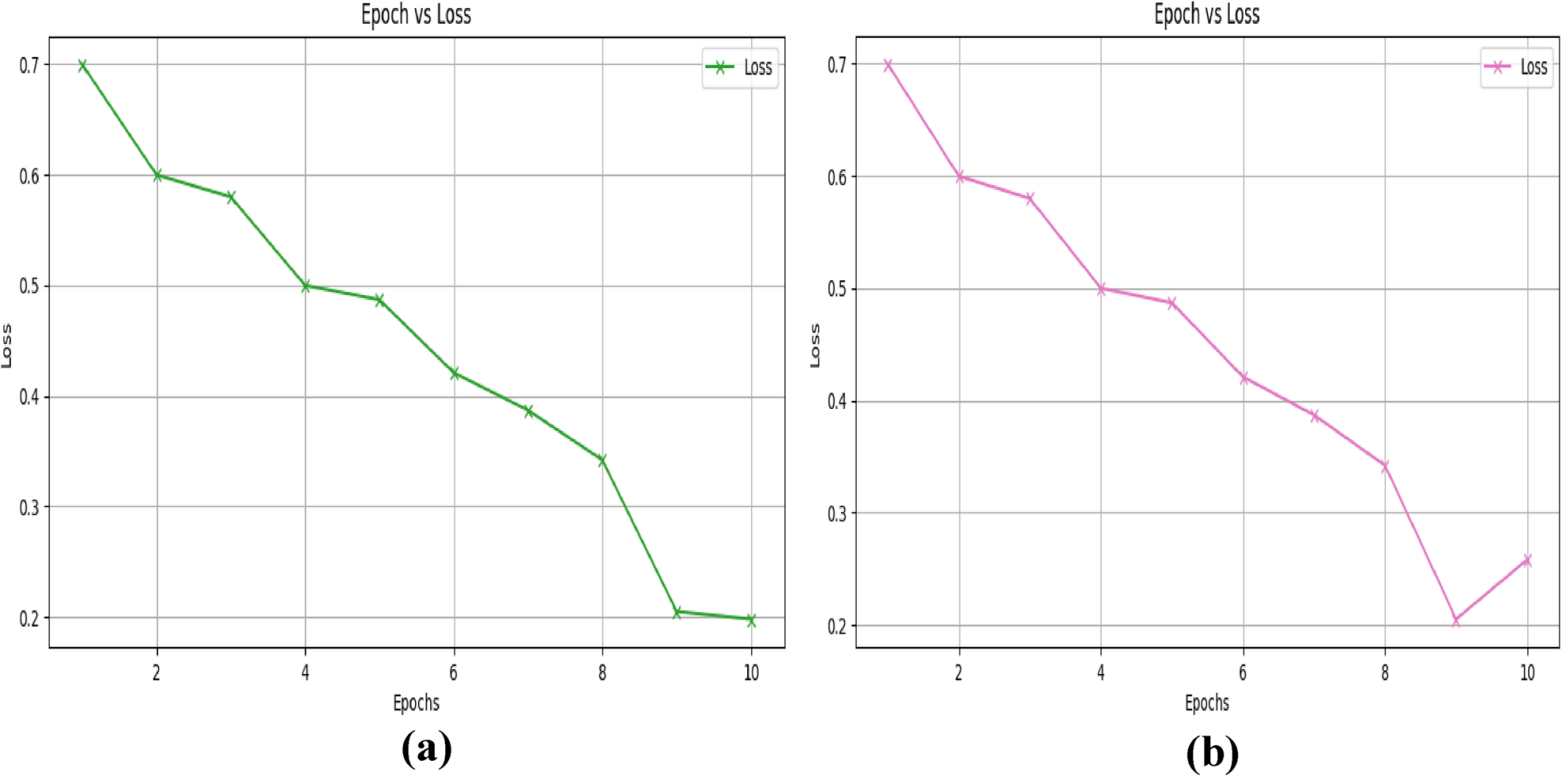
Epoch- loss graph for the KDD-Cup 99 dataset (**a**) training and (**b**) validation.

The accuracy-epoch graph for training and validation, which uses the NSL-KDD dataset and is displayed in figures 7(a) and (b), shows how accuracy progressively rises with increasing epoch. Figure 8 (a) and (b), which use the NSL-KDD dataset, illustrate the loss-epoch relationship for training and validation.

**Fig. 9:**
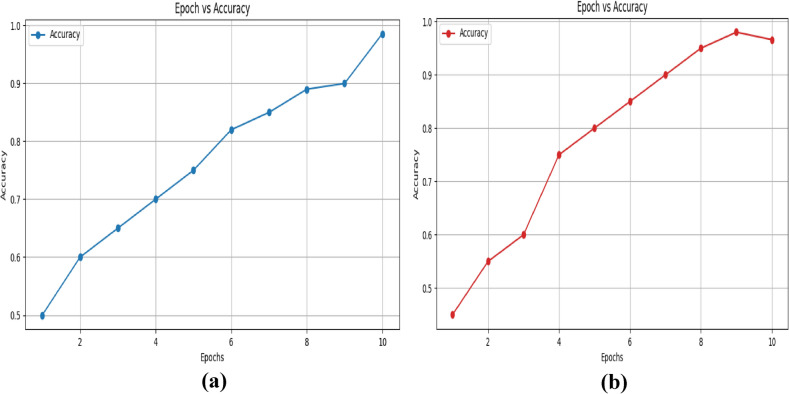
Epoch- accuracy graph for the UNSW-NB 15 dataset (**a**) training and (**b**) validation.

**Fig. 10:**
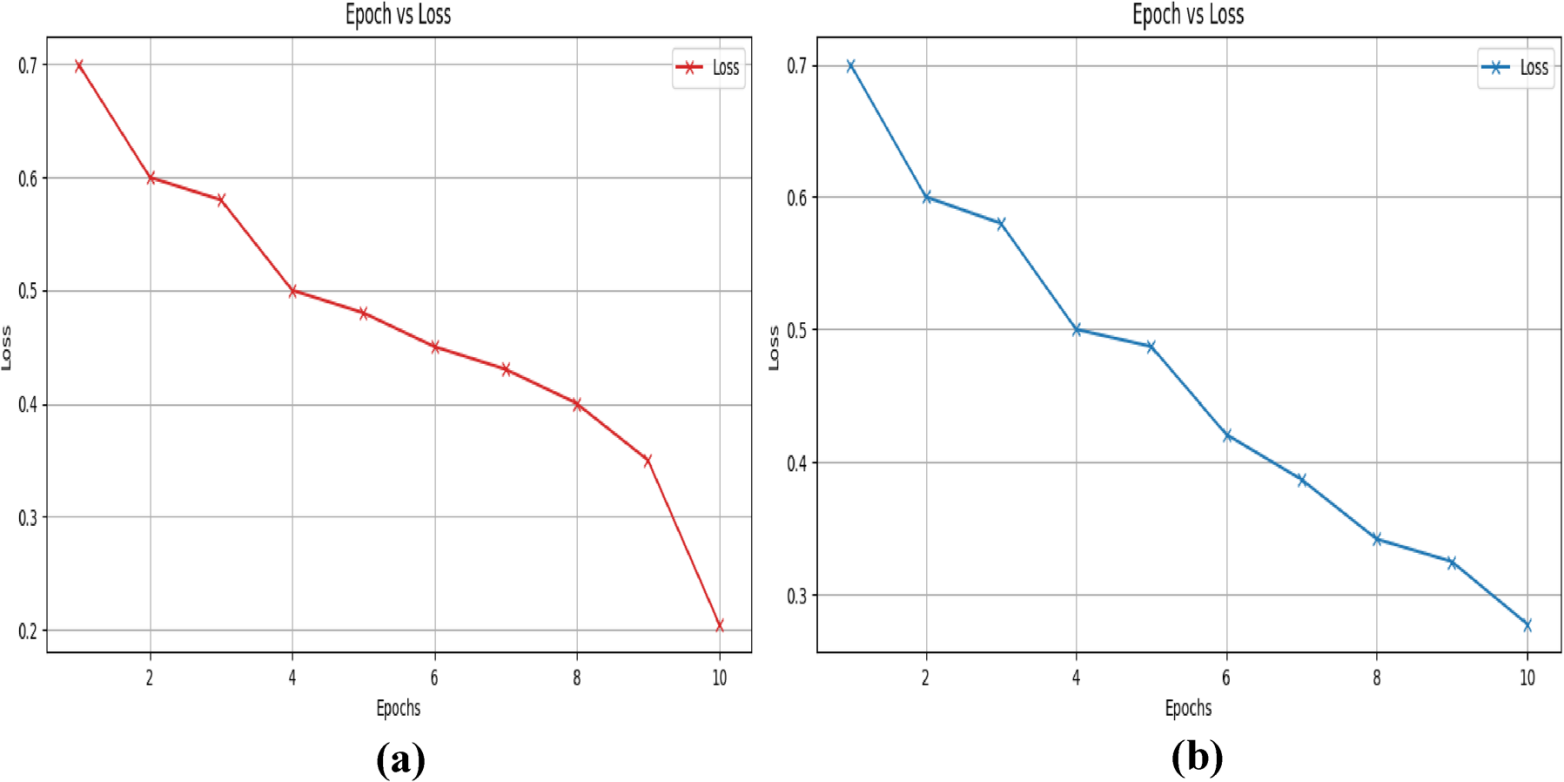
Epoch- loss graph for the UNSW-NB 15 dataset (**a**) training and (**b**) validation.

They demonstrate how the loss value falls as the epoch increases. Similarly, we used the UNSW-NB 15 dataset to analyze the accuracy-epoch graph and loss-epoch graph of the suggested technique in figures 9 and 10.

### Comparative analysis results

To demonstrate the effectiveness of the suggested strategy, we contrast our approach with a number of others. We’ve examined two different scenarios: one involves analyzing intrusion detection techniques and the other involves evaluating secure data sharing method.

#### Comparative analysis of intrusion detection system

This section compares the effectiveness of our proposed ECaps-Net against Capsule Network, LSTM, and SVM using UNSW-NB 15 and KDD Cup 99 datasets. The contrast is examined utilizing a count of metrics, including as f-score, recall, precision, and sensitivity. Capsule Network, LSTM, and SVM are chosen for this comparison due to their widespread use in intrusion detection and their unique strengths in detecting various kinds of intrusions. This comparison clearly demonstrates the advantages of the proposed ECaps-Net.

Table 3 depicts the intrusion detection comparative analysis of suggested method with other methods utilizing KDD Cup 99 dataset. The suggested ECaps-Net model attained a higher accuracy rate of 98.90%, significantly outperformed SVM by 9.34%, LSTM by 5.66%, and CapsNet by 2.36%. The precision of proposed ECaps-Net is 98.78%, which is 9.35% higher than SVM, 5.52% higher than LSTM, and 2.27% higher than CapsNet. The recall of proposed ECaps-Net is 98.65%, significantly outperformed SVM by 9.42%, LSTM by 5.47%, and CapsNet by 2.17%. The proposed ECaps-Net achieved a sensitivity of 98.54%, which is 9.25% higher than SVM, 5.38% higher than LSTM, and 2.31% higher than CapsNet. The F-score of ECaps-Net is 98.45%, which outperformed SVM by 9.91%, LSTM by 5.20%, and CapsNet by 2.33%. The proposed ECaps-Net model outperformed all other methods across all metrics. These improvements of proposed ECaps-Net are attributed to the integration of SE block into the conventional Caps-Net, which effectively highlights important features, reducing misclassification and enhancing accuracy and overall performance of the intrusion detection system.

**Table 3:** Performance analysis of intrusion detection system using kdd cup-99 dataset.

Methods	Accuracy (%)	Precision (%)	Recall (%)	Sensitivity (%)	F-score (%)
SVM	89.56	89.43	89.23	89.29	88.54
LSTM	93.24	93.26	93.18	93.16	93.25
CapsNet	96.54	96.51	96.48	96.23	96.12
Proposed ECapsNet	98.90	98.78	98.65	98.54	98.45

Table 4 shows the intrusion detection comparative analysis of proposed and existing methods using UNSW-NB 15 dataset. The suggested ECaps-Net method attained higher accuracy rate of 98.78%, significantly outperformed SVM by 9.33%, LSTM by 5.60%, and CapsNet by 2.46%. The precision of proposed ECaps-Net is 98.74%, which is 9.36% higher than SVM, 5.58% higher than LSTM, and 2.29% higher than CapsNet. The recall of proposed ECaps-Net is 98.54%, significantly outperformed SVM by 9.36%, LSTM by 5.39%, and CapsNet by 2.21%. The sensitivity of proposed ECaps-Net is 98.43%, which is 9.20% higher than SVM, 5.31% higher than LSTM, and 2.22% higher than CapsNet. The F-score of ECaps-Net is 98.32%, which outperformed SVM by 9.96%, LSTM by 5.18% and CapsNet by 2.26%. The proposed ECaps-Net model consistently outperformed existing methods across all metrics. By integrating the SE block into the conventional CapsNet, which dynamically recalibrates feature maps by giving more weights to significant features and decreasing less relevant ones, the suggested ECaps-Net performs better than the original CapsNet. This results in more accurate and dependable intrusion detection in IoT edge computing environments.

**Table 4:** performance analysis of intrusion detection system using unsw-nb 15 dataset.

Methods	Accuracy (%)	Precision (%)	Recall (%)	Sensitivity (%)	F-score (%)
SVM	89.45	89.38	89.18	89.23	88.36
LSTM	93.18	93.16	93.15	93.12	93.14
CapsNet	96.32	96.45	96.33	96.21	96.06
Proposed ECapsNet	98.78	98.74	98.54	98.43	98.32

#### Cross-validation analysis

A five-fold cross-validation approach is used with the KDD Cup 99 and UNSW-NB 15 datasets to ensure the robustness and generalization capacity of the suggested ECaps-Net model. The dataset was divided into five equal-sized folds using this method. For every iteration, four folds were used for training, while the fifth fold was used for testing. This process was carried out five times, with each fold serving as the test set once. To provide a reliable approximation of the model’s capacity for classification, the performance indicators were then averaged across all folds. The validation procedure guarantees that the ECaps-Net is resilient across different data subsets and lessens bias from a single train-test split.

The proposed ECaps-Net’s five-fold cross-validation results on the UNSW-NB 15 and KDD Cup 99 datasets are shown in Table 5. The results showed that the suggested model performed well on both datasets across all folds. Accuracy ranged from 98.88% to 98.95% with a mean of 98.90 ± 0.04 on the KDD Cup 99 dataset, and from 98.68% to 98.90% with a mean of 98.80 ± 0.09 on the UNSW-NB 15 dataset. Both datasets’ precision, recall, F-score, and sensitivity values remained stable over folds, demonstrating the ECaps-Net model’s high stability, generalizability, and robustness.

**Table 5:** performance evaluation of ecaps-net using five-fold cross-validation.

Datasets	Evaluation Metrics	Fold-1	Fold-2	Fold-3	Fold-4	Fold-5	Mean ± SD
KDD Cup 99 dataset	Accuracy	98.85	98.92	98.88	98.95	98.88	98.90 ± 0.04
	Precision	98.72	98.80	98.76	98.83	98.78	98.78 ± 0.04
	Recall	98.60	98.68	98.63	98.70	98.65	98.65 ± 0.04
	Sensitivity	98.50	98.58	98.54	98.60	98.55	98.55 ± 0.04
	F-Score	98.45	98.50	98.47	98.50	98.48	98.48 ± 0.03
UNSW-NB 15 dataset	Accuracy	98.82	98.75	98.68	98.90	98.85	98.80 ± 0.09
	Precision	98.76	98.70	98.68	98.78	98.74	98.73 ± 0.05
	Recall	98.62	98.50	98.55	98.65	98.58	98.58 ± 0.06
	Sensitivity	98.55	98.45	98.50	98.58	98.52	98.52 ± 0.05
	F-Score	98.42	98.38	98.35	98.45	98.40	98.40 ± 0.04

#### Comparative analysis of secure data transmission framework

This section compares the effectiveness of our proposed Merkel Damgard Cryptographic algorithm with other existing algorithm such as Miyaguchi-Preneel Cryptographic algorithm and SHA-256 using KDD Cup-99 and UNSW-NB 15 datasets in terms of confidentiality rate, processing time and data integrity rate.

Table 6 shows the comparison of safe data sharing in IoT edge computing environment of suggested algorithm with other algorithms utilizing KDD Cup 99 dataset. Analyzing the effectiveness of existing algorithms for secure data transmission, the proposed Merkel Damgard cryptographic algorithm outperformed other existing algorithms. The proposed algorithm achieved a higher confidentiality rate of 98.20%, reflects its superior ability to prevent unauthorized access or data leakage during transmission. In terms of confidentiality rate, the developed algorithm displays a notable advancement over the existing algorithms. Compared to SHA-256 and Miyaguchi-Preneel Cryptographic algorithm, the proposed algorithm improves confidentiality rate by 4.9% and 2.92%, respectively. The reason for achieving higher data confidentiality is primarily due to the Merkle–Damgård cryptographic algorithm, which forms a chain of users by linking blocks through one-way hash function. Each hash is dependent on the previous block. The hash-based data transmission is performed to access the data only by the authorized entity and it avoids the unauthorized entity. In terms of data integrity rate, the proposed algorithm achieved a higher data integrity rate of 97.90%, outperforming SHA-256 by 5.05% and outperforming Miyaguchi-Preneel Cryptographic algorithm by 2.78%. This is because the reason for achieving a higher data integrity rate is attributing to the one-way compression function in the Merkle–Damgård cryptographic algorithm. The compression function generates the fixed size of the output while giving the fixed size of the input. If any changes in the input data cause a severe change in the hash value. This helps to easily identify any alteration in the input data. These results illustrated the proposed algorithm maintaining data confidentiality and integrity. The processing time is crucial in real-time IoT edge computing environments, the proposed algorithm takes processing time is 2 mins, the processing time of existing algorithms such as SHA-256 is 3 m 56 s and Miyaguchi-Preneel Cryptographic algorithm is 2 m 28s. The result indicated the proposed method requires less time for transmitting the data into the server. The proposed algorithm outperforms other algorithm by achieving higher confidentiality and integrity rates with faster processing. The proposed algorithm more effective in securing data and also more efficient in processing time, the suggested approach offered a reliable way to transmit data securely in IoT edge computing settings.

**Table 6:** Performance analysis of secure data transmission using kdd cup 99 dataset.

**Algorithms**	**Confidentiality rate**	**Data integrity rate**	**Processing time**
SHA-256	93.24%	92.85%	3 m 56s
Miyaguchi-Preneel Cryptographic algorithm	95.28%	95.12%	2 m 28s
Proposed Merkle-Damgard Cryptographic algorithm	**98.20%**	**97.90%**	**2m**

Similarly, table 7 shows the comparison of secure data sharing in IoT edge computing environment using UNSW-NB 15 dataset. The proposed method Merkel Damgard Cryptographic algorithm attained a higher confidentiality rate of 97.28%, indicating strong protection against unauthorized access and data leakage. The proposed algorithm outperforming the existing algorithms, 5.43% improvements over SHA-256, and 3.10% improvements over Miyaguchi-Preneel Cryptographic algorithm. The proposed method Merkel Damgard Cryptographic algorithm attained a higher data integrity rate reached 96.98%**,** outperforming SHA-256 by 5.54% and outperforming Miyaguchi-Preneel Cryptographic algorithm by 2.98%. This high integrity rate demonstrates the algorithm’s ability to detect alterations in the data during transmission. These results highlight the proposed algorithm maintaining data integrity and confidentiality. The processing time of the suggested algorithm is 2 m 10 s, while the processing time of the existing method such as SHA-256 is 3 m 58 s and Miyaguchi-Preneel Cryptographic algorithm is 2 m 8s. The result indicated that the proposed method requires less time. The proposed algorithm outperforms other algorithm by achieving higher confidentiality and integrity rates with faster processing. The proposed algorithm demonstrated it effectiveness in secure data transmission in IoT environments.

**Table 7:** Performance analysis of secure data transmission using UNSW-nb 15 dataset.

Algorithms	Confidentiality rate	Data integrity rate	Processing time
SHA-256	92.27%	91.89%	3 m 58s
Miyaguchi-preneel cryptographic algorithm	94.18%	94.17%	2 m 38s
Proposed merkle-damgard cryptographic algorithm	97.28%	96.98%	2 m 10s

##### Performance evaluation of merkle-damgård cryptographic algorithm under different network loads

To evaluate the efficiency of the proposed Merkle-Damgård cryptographic algorithm in IoT edge environments, we compare the proposed Merkle-Damgård cryptographic algorithm with existing SHA-3 and BLAKE-2 algorithms in terms of hash computation time under three network load scenarios: low (100 KB/s), medium (1 MB/s), and high (10 MB/s).

Table 8 presents the hash computation times for proposed Merkle-Damgård cryptographic algorithm with existing SHA-3 and BLAKE2 algorithms in terms of hash computation time under varying network loads. The results indicate that the proposed Merkle-Damgård algorithm consistently achieves minimum hash computation times across all network loads, demonstrating its suitability for real-time IoT-edge data transmission. The computation time slightly increases with higher network loads, which is expected due to the larger volume of data processed. Compared with SHA-3 and BLAKE2, the proposed algorithm maintains low hash computation times while providing secure hashing, making it appropriate for resource-constrained IoT environments.

**Table 8:** Hash computation time under different network loads for proposed vs. existing algorithms.

**Network load**	**Proposed merkle-damgård**	**BLAKE-2**	**SHA-3**
Low (100 KB/s)	1.25	1.22	1.38
Medium (1 MB/s)	3.12	3.05	3.45
High (10 MB/s)	12.40	11.98	13.75

#### Comparative analysis of proposed ECapsNet with Merkle–Damgåd cryptography

To prove the effectiveness of the suggested framework, we compared it against different IDS frameworks and cryptography algorithms based on latency, throughput, and energy consumption. Table 9 shows the comparative analysis results of the suggested ECapsNet+Merkle–Damgård framework with ECapsNet+BLAKE3, ECapsNet+SHA-3, Vision Transformer (ViT)+Merkle–Damgård, and Swin Transformer(ST)+Merkle–Damgård.

**Table 9:** Comparison of proposed framework with different cryptographic algorithms and ids models.

Model	Latency (ms)	Throughput (Mbps)	Energy Consumption (J)
Proposed ECapsNet + Merkle–Damgård	12	480	1.8
ECapsNet + BLAKE3	13	470	1.9
ECapsNet + SHA-3	14	460	2.0
ViT+Merkle–Damgård	25	370	3.9
ST + Merkle–Damgård	28	350	4.2

From Table 9, that the proposed framework significantly outperforms the other frameworks, with achieves lowest latency of 12 ms and energy consumption of 1.8 J, with the maximum throughput of 480 Mbps. in comparison, ECapsNet with BLAKE3 and SHA-3 has slightly higher latency and energy consumption, while transformer-based IDS models (ViT and ST) have higher latency and energy consumption, with lower throughput. These findings suggests that the proposed framework is efficient when E-CapsNet coupled with Merkle–Damgård cryptographic algorithm, and thus more suitable for real-time intrusion detection and secure data transmission in IoT edge computing.

### Comparison with published works

To illustrate the efficiency of the suggested approach, we contrast our work with previously published research^16–23^. Most of these existing methods have been applied in the IoT edge computing environments for intrusion detection.

A detailed review of these works is provided in the literature survey section, highlighting their methodologies, performance, and limitations. Table 10 shows the comparative analysis of our proposed method with previously published studies. Our proposed ECapsNet integrated with the Merkle–Damgård Cryptographic algorithm achieved superior performance across all evaluation metrics on the KDD-CUP 99 and UNSW-NB 15 datasets. Examining Table 6, we found that our suggested approach produced the best accuracy, 98.90% in the KDD-CUP 99 and 98.78% in the UNSW-NB 15 dataset. In contrast, the accuracy rates reported in^16–23^ were 83.09%, 93.96%, 97.89%, 84.86%, 98%, 90.25%, 98.13%, and 96.02%, respectively. Additionally, our method obtained the highest precision and recall of 98.74% and 98.78%, and 98.54% and 98.65% on UNSW-NB 15 and KDD-CUP 99 datasets, respectively. In terms of F-score, the proposed method achieved highest F-scores of 98.45% and 98.32%, on UNSW-NB 15 and KDD-CUP 99 datasets, respectively. Our approach surpassed current approaches by producing better results across various metrics, demonstrating its efficacy in accurately classifying the data. These significant improvements can be attributed to our proposed ECapsNet, the integration of the SE block in the traditional CapsNet. This design emphasizes important features while suppressing irrelevant information, leading to more accurate and reliable intrusion detection in IoT edge computing environments. Moreover, unlike previous studies that primarily concentrate on intrusion detection, our approach also addresses secure data transmission, which is a critical requirement in IoT environments. The blockchain-based Merkle-Damgard Cryptographic algorithm demonstrated it effectiveness in secure data transmission in IoT edge computing environments. The classified data are sent to the server in the form of hash value, preserving confidentiality and integrity while minimizing processing time. Besides, the transactions of blockchain are operated independently without the requirement of a third party; the data transmission is both secure and efficient. The proposed method not only achieves state-of-the-art performance in intrusion detection but also ensures secure, fast, and trustworthy data transmission in IoT edge computing environments, thereby outperforming existing approaches in both detection accuracy and security efficiency.

**Table 10:** Performance comparison of proposed work vs. published works.

Ref. No	Methods	Datasets	Performance Analysis				Limitations
			Accuracy (%)	Precision (%)	Recall (%)	F-score (%)	
^16^	ESOML	UNSW-NB15	83.09	82.48	82.50	83.08	Low accuracy in detecting intrusions
^17^	XGBoost-TCN	AWID	93.96	64.36	66.22	65.27	High computational efficiency
^18^	HBFL	IoT dataset	97.89	-	-	94.80	Does not detect sophisticated adversaries
^19^	BiGRU-DNN	NSLKDD, UNSWNB15, CICIDS2017	84.86	84.73	85.21	84.88	High computational efficiency
^20^	FL	IoTID20, IoT-23, N-BaIoT	98	-	-	-	Low processing power
^21^	Meta-AdaboostM1 algorithm	UNSW-NB 15	90.25	86.14	94.59	86.95	Low efficiency and scalability
^22^	PCCNN	NSL-KDD	98.13	-	-	-	High computational time
^23^	BFLIDS, CNN, BiLSTM	NSL-KDD	96.02	96	95	96	Difficult to detect complex patterns
Ours	Proposed ECapsNet with Blockchain-based Merkel Damgard Cryptographic algorithm	KDD-CUP 99	98.90	98.78	98.65	98.45	
		UNSW-NB 15	98.78	98.74	98.54	98.32	

### Security analysis

Secure and attack-proof data transmission is crucial in IoT edge computing environments. The developed framework integrates a permissioned Hyperledger Fabric blockchain with the Merkle–Damgård hashing algorithm, providing data integrity, confidentiality, and resistance to malicious attacks. The blockchain network may also be susceptible to attacks such as 51% attacks, where an attacker controlling the majority of nodes may attempt ledger tampering; Sybil attacks, which involve multiple fake identities used to influence consensus; data poisoning, which is employed to inject malicious data; and key compromise, where private keys are compromised.

In the proposed system, these attacks are managed as follows: the permissioned Fabric blockchain network restricts participation to authenticated devices, reducing the risk of Sybil attacks and illicit access. The PBFT consensus protocol ensures consensus among participating nodes, precluding unilateral tampering and mitigating against 51% attacks. The Merkle–Damgård hash chaining cryptographically links blocks together, hence any modification can be detected in real-time, protecting against data poisoning. Moreover, cryptographic hashing of confidential information ensures that information remains secure even when communications over the network are diverted, reducing the impact of key compromise. By following this strategy, the framework ensures data integrity, confidentiality, and resistance to common blockchain attacks, thereby making the framework suitable for secure IoT edge computing.

## Conclusion

This study presented a novel framework for secure data transmission in IoT edge computing environments by integrated an ECaps-Net with Blockchain-based Merkle-Damgard Cryptographic algorithm. The intrusion dataset was preprocessed by unnecessary data removal and applying normalization to enhance data quality. The proposed ECaps-Net was designed to classify the pre-processed data as normal or intruded. By integrated a SE block into the traditional CapsNet, ECaps-Net was able to enhance classification accuracy by highlighting important features and suppressing irrelevant features. Blockchain technology was utilized for converting the classified normal data into blocks. A one-way compression function based on the Merkle–Damgård cryptographic algorithm was used to generate fixed length hash for each block. The suggested ECaps-Net based IDS system outperformed existing methods, achieved remarkable accuracy of 98.90% and 98.78%, precision of 98.78% and 98.74%, recall of 98.65% and 98.54%, sensitivity of 98.54% and 98.43%, and F-score of 98.45% and 98.32% on the KDD-CUP 99 and UNSW-NB 15 datasets, respectively. Furthermore, the Blockchain-based Merkle-Damgard cryptographic algorithm outperformed existing algorithms by attaining higher data integrity of 97.90% and 96.98%, and confidentiality rate of 98.20% and 97.28%, while reduced processing time of 2 m and 2 m 10 s on KDD-CUP99 and UNSW-NB 15 datasets, respectively. This integrated framework offered a robust solution for intrusion detection and secure data transmission in IoT edge computing environments. Future research could investigate the integration of FL to further improve both security and privacy. Additionally, the blockchain framework can be extended to support zero-trust architectures and multi-factor authentication mechanisms to enhance access control and trust management in IoT edge environments. This would further strengthen data confidentiality and ensure that only verified entities participate in the blockchain network. Moreover, the proposed framework will be deployed and tested on real-time heterogeneous IoT data to evaluate its scalability, adaptability, and practical applicability.

## Data Availability

The KDD-Cup 99 and the UNSW-NB 15 datasets are analysed during the current study are publicly available at http://kdd.ics.uci.edu/databases/kddcup99/kddcup99.html and https://research.unsw.edu.au/projects/unsw-nb15-dataset
